# The psychosocial factors influencing paediatric kidney transplantation access, their outcomes and the patient and family’s perceived quality of life: a systematic review and meta-analysis

**DOI:** 10.1007/s00467-025-07058-9

**Published:** 2025-12-10

**Authors:** Ji Soo Kim, Camille Laroche, Thozama Siyotula, Stephen D. Marks, Jo Wray

**Affiliations:** 1 Department of Paediatric Nephrology, Great Ormond Street Hospital for Children NHS Foundation Trust, Great Ormond Street, London, WC1N 3JH UK; 2 NIHR Great Ormond Street Hospital Biomedical Research Centre, Great Ormond Street Institute of Child Health, University College London, London, UK; 3 Department of Paediatric Nephrology, Centre Hospitalier Universitaire Sainte-Justine, Montreal, Canada; 4 Division of Paediatric Surgery, Red Cross War Memorial Children’s Hospital, University of Cape Town, Cape Town, South Africa; 5 Centre for Outcomes and Experience Research in Children’s Health, Illness and Disability, Great Ormond Street Hospital for Children NHS Foundation Trust, London, UK

**Keywords:** Psychosocial factors, Kidney transplantation, Quality of life

## Abstract

Kidney transplantation is the gold standard treatment for children and young people (CYP) with stage 5 chronic kidney disease (CKD). However, psychosocial and structural factors including race, socioeconomic status and family context can delay or limit access to transplantation. This systematic review explores the breadth of psychological, social and societal factors influencing transplant access, outcomes and quality of life. We included peer-reviewed quantitative, qualitative and mixed-method studies with primary data. Medline, PsychInfo, CINAHL and Web of Science were searched for papers published in English between January 1964 and November 2022. A total of 66 studies (52 quantitative, 10 qualitative and 4 mixed-methods) were identified and analysed. A wide breadth of psychosocial factors were identified: mental health, adjustment to CKD, non-adherence, beliefs, motivation, cognitive status, family structure, family relationships, trust in or relationship with healthcare professionals, health insurance, infrastructure, education, geography, socioeconomic status (SES), social support, housing, religion or culture, national or regional wealth, migrant status-driven or racialised health inequities and kidney allocation policies. Among these studies, 13 qualified for meta-analysis regarding SES and race. CYP experiencing lower SES as a potential stressor were more likely to access deceased donor transplantation, less likely to access pre-emptive transplantation and more likely to experience allograft failure. CYP from racially minoritised groups had reduced access to living donor and pre-emptive transplantation, and higher rates of allograft loss. These inequities were often mediated by structural racism, implicit bias, allocation policies and overlapping systems of disadvantage. There is a wide breadth of psychosocial factors that influence the lived experience of CKD and access to and outcomes after transplantation. Race, as a proxy for racism, interacts with SES, geography and other social determinants through an intersectional process that deepens inequities. Some factors are modifiable at the clinical or family level, while others require systemic change. Addressing these inequities will require multi-tiered strategies, from clinician education on bias to reforms in allocation policy and broader social structures. Further studies are needed to better understand the complex relationship between these psychosocial factors and kidney transplantation outcomes. Prospero Registration. number CRD42020210506.

**Graphical Abstract:**

**Figure:**
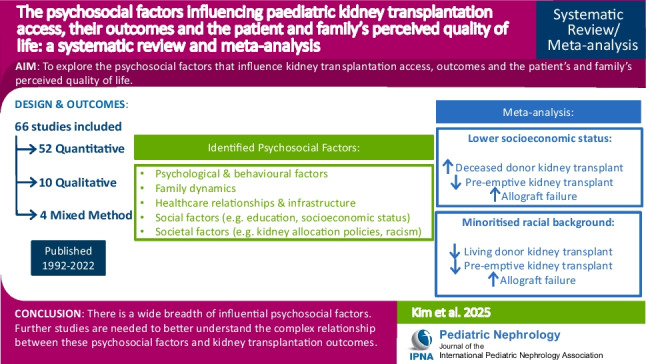
A higher resolution version of the Graphical abstract is available as Supplementary information

**Supplementary information:**

The online version contains supplementary material available at 10.1007/s00467-025-07058-9.

## Background

Kidney replacement therapies (KRT), including dialysis and/or kidney transplantation may not be curative but offer life-sustaining intervention for paediatric patients with stage 5 chronic kidney disease (CKD). Kidney transplantation is seen as the gold standard KRT for patients with stage 5 CKD [1]. Kidney transplant recipients are less likely to experience cardiovascular complications and have a better survival rate and quality of life (QoL) than children and young people on dialysis [2, 3]. Evidence has also shown better health outcomes in those who spent less time on dialysis before transplantation and received their transplant from a living donor [4, 5].

However, not every medically eligible CYP undergoes a kidney transplant. Huge variation in pre-, peri- and post-transplant practices have been described internationally [6–9]. ‘Psychosocial factors’ have been reported to contribute to 19% of kidney transplantation delays for CYP in the UK [10]. However, it is not clear what these psychosocial factors are.

The aim of this systematic review was to explore the range of psychosocial factors influencing access to paediatric kidney transplant and recipients’ subsequent kidney transplant outcomes.

## Methods

### Data sources and search strategy

This review was registered with the International Prospective Register for Systematic Reviews (PROSPERO: CRD42020210506 [11]) and reported as per the Preferred Reporting Items for Systematic Reviews and Meta-Analyses ‘PRISMA’ and Enhancing Transparency in Reporting the synthesis of Qualitative research: ‘ENTREQ’ checklists [12, 13]. Medline, PsychInfo, CINAHL and Web of Science databases were searched. The search strategy used a combination of Medical Subject Headings (MeSH) and relevant controlled vocabulary (Supplemental material 1). Reference lists in publications generated by the search were hand-searched, and experts in the field were consulted to ensure every relevant study was captured.

### Study eligibility criteria

#### Phenomena of interest

Psychological or social factors relevant to the following:

- Access to paediatric kidney transplantation
- Transplant eligibility decision-making by clinicians
- Post-transplant outcomes
- The lived experience of families with stage 5 CKD

Study outcomes of interest included, but were not limited to the following:

- Description of the above psychological or social factors
- Time to receive transplant
- Time to kidney allograft failure
- Mortality rate while awaiting transplantation
- Health-related QoL
- Time to kidney allograft failure or deterioration

Inclusion criteria:

- Participants in studies were either patients aged < 18 years old and/or their caregivers and/or their healthcare professionals
- Studies with participants aged < 18 years old with any of these characteristics: not on KRT but diagnosed with stage 5 CKD, or on chronic KRT (dialysis or transplantation)
- Studies conducted in high- or middle-income countries
- Published in the English language
- Published between January 1964 and November 2022
- All study designs (quantitative, qualitative and mixed-method studies)

Exclusion criteria:

- Editorial commentaries, reviews and conference abstracts
- Studies including pregnant participants, multi-organ transplant recipients or participants without a kidney condition.

Two reviewers (JSK and JW) independently screened the abstracts of identified studies against the eligibility criteria with a third reviewer (SDM) resolving conflicts. The full texts of initially included studies were then independently screened by JSK and SDM against the eligibility criteria with JW resolving disagreements. JSK then extracted and tabulated data from included studies, which were reviewed with SDM and JW.

### Study appraisal and synthesis methods

Since this review included quantitative, qualitative and mixed-method studies, the Mixed Methods Appraisal Tool (MMAT) [14] was used (Supplemental material 2). Conventionally the MMAT does not provide an overall score, but for purposes of summarising this review, a breakdown and an overall score of 0 to 10 was assigned. Using the MMAT, two reviewers (JSK and CL) independently reviewed the quality of the included studies and discussed to reach consensus. Any remaining disagreements were resolved with reviewers JW and SDM.

A meta-analysis was performed on studies where the same outcome measures were used in three or more RCT or quantitative non-randomised studies for a particular psychosocial factor. Access to kidney transplantation was defined as the successful receipt of kidney transplant in any time period via any of these types: pre-emptive (PKT) and/or deceased-donor (DDKT) or living-donor kidney transplant (LDKT). As each type of transplant depends on differing factors that influence recipient circumstances and donor availability, meta-analyses were performed for overall access to transplantation as well as for each transplant type. Outcome of kidney transplantation was defined by the 5-year renal allograft survival rate. Therefore, we excluded studies (1) with less than 5-year renal allograft survival data [15, 16], (2) where the odds ratio could not be calculated using the available study data [17–20] or (3) which measured other outcomes (e.g. death or psychological factors) [3, 21–−31]. Mantel–Haeszel odds ratios with the assumption of random-effects at a 95% confidence interval were used to account for the statistical heterogeneity between the studies (e.g. set in different countries with different healthcare policies that may impact transplant access). To harmonise the different definitions of socioeconomic status (SES) between studies (i.e. binary or quintiles categories), the highest SES category was analysed against the lowest SES category. In all studies that examined racialised health inequities, comparison groups were broadly based on perceived racial identity or shared cultural identity and heritage (Hispanic, Indigenous). The racially centred group was White and the racially minoritised groups were Black, Hispanic or Indigenous. RevMan® software (version 5.4.1, The Cochrane Collaboration) was used to perform the meta-analyses.

Given the mixture of thick and thin data in the selected qualitative studies, they were analysed through thematic synthesis [32] using Nvivo Software for data management (13th edition, Lumivero). Thematic synthesis is a qualitative research method used in systematic reviews to identify themes across multiple studies. Data for extraction and coding included both direct quotes and key concepts from the studies. One reviewer (JSK) undertook the initial inductive coding of the text and developed the descriptive themes. These were independently reviewed by JW and SDM and then discussed between the three reviewers (JSK, JW and SDM) before generating the final analytical themes through consensus. For studies where meta-analysis or thematic synthesis could not be performed, a narrative synthesis was performed as per SWiM guidelines [33]. An integrated analysis was then performed where relevant psychosocial factors were tabulated against findings from the meta-analyses, thematic synthesis and narrative description of the studies to review for consensus between the studies.

## Results

**Fig. 1:**
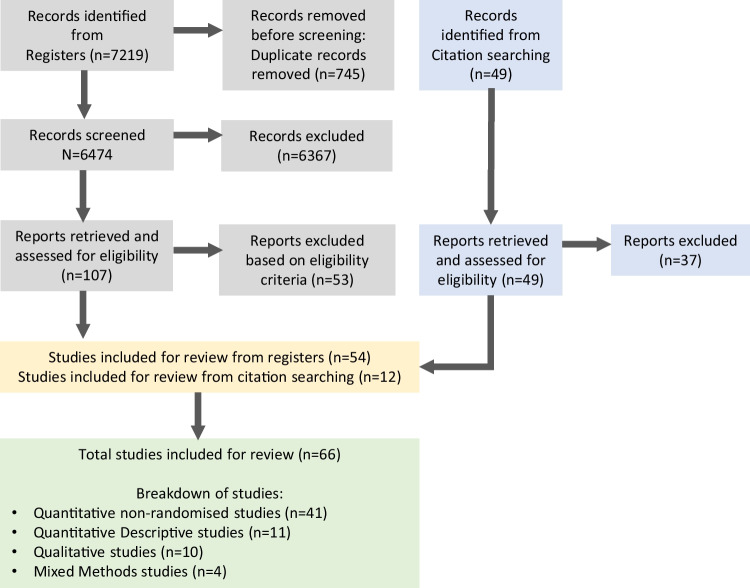
Flow chart of studies included

The initial database search before screening yielded 7268 papers, and 66 were included in the final review, the majority of which were quantitative non-randomised studies (*n* = 41) (Fig. 1).

Table 1 summarises all study characteristics including setting, population, study design and MMAT scores. Publication dates of included studies spanned from 1992 to 2022.

**Table 1:**
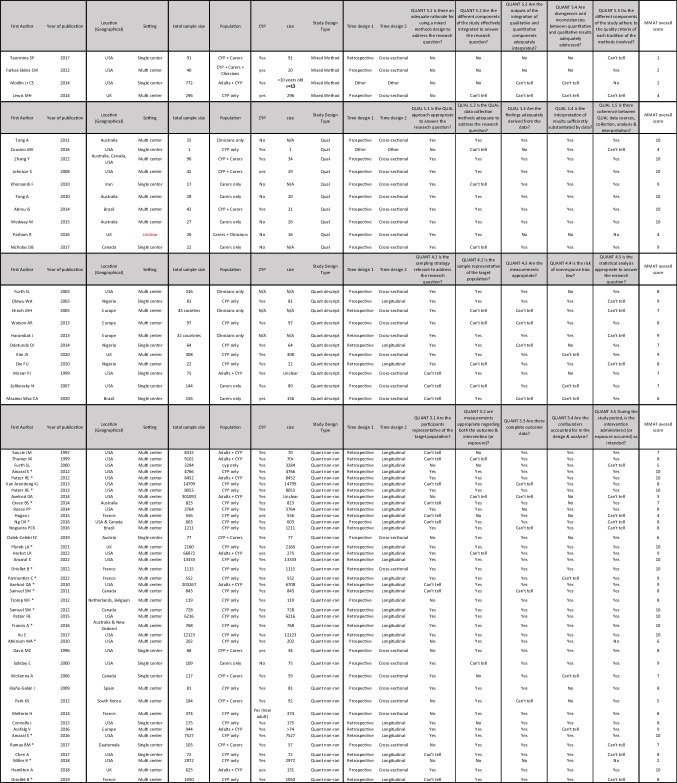
Summary of all study characteristics including MMAT scores

### Quality appraisal

No studies were excluded from analysis based on MMAT score, as the purpose of this review was to explore and describe the full breadth of possible psychosocial factors that influence access to and outcomes of paediatric kidney transplantation. There was variability across study designs in terms of study quality. All four studies categorised as mixed method scored 4 or less overall, due to the lack of reported rationale for using a mixed method design and integration of the different components to answer the study’s research question. The ten qualitative studies included for review scored between 4 and 10. The two qualitative studies with scores of 4 were unclear as to how the findings were adequately derived from data. Scores for the 11 quantitative descriptive studies ranged from 6 to 9. There were 41 quantitative non-randomized studies and although scores ranged from 2 to 10, 30 (73%) studies scored 8 or above.

### Summary of findings

Given the breadth of international coverage from included studies, a broad spectrum of medical infrastructure was represented. Supplemental material 3 shows validated questionnaires utilised by studies examining mental health, QoL or other related factors. Only 13 studies were eligible for meta-analysis comparing different socioeconomic status (SES) groups or racial groups against the likelihood of accessing a transplant or allograft failure.

**Fig. 2:**
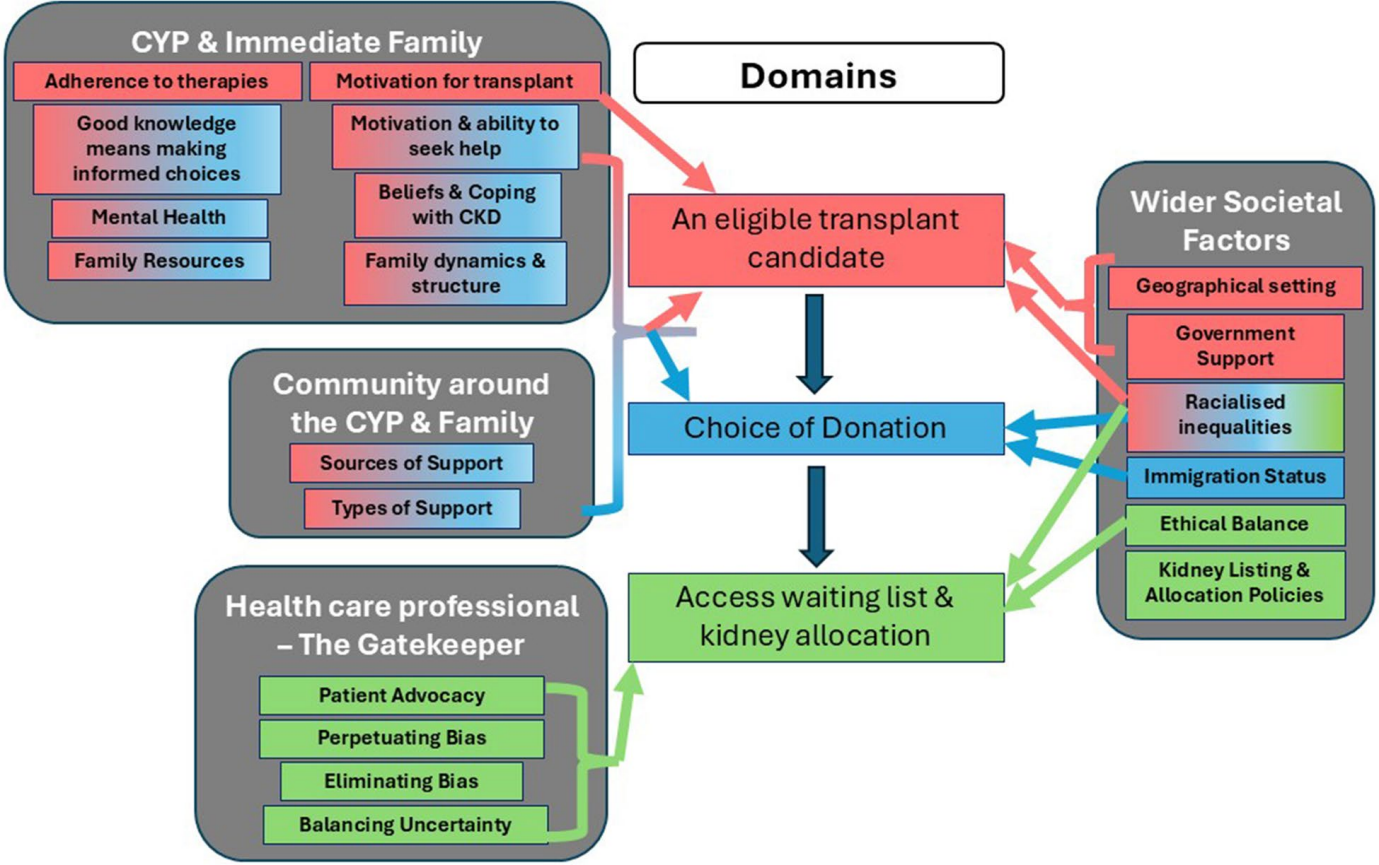
Thematic synthesis of qualitative themes

The thematic synthesis of the qualitative studies and qualitative content of the mixed method studies generated three broad, overarching domains with four themes and 20 subthemes, shown in Fig. 2. These three domains were (1) ‘An eligible transplant candidate’, (2) ‘Choice of Donation’ (i.e. DDKT, LDKT and PKT) and (3) ‘Access waiting list and kidney allocation’.

Factors which influenced whether a CYP was perceived as eligible for transplantation included how well the CYP and family adhered to therapies, how motivated they were for transplantation and wider societal factors such as their geographical setting or availability of government support [34–42]. Although immigration status of the CYP held less importance to clinicians and families in terms of their transplant candidacy [36], immigration status of potential living donors influenced their decision to donate [40]. The factors influencing the CYP’s access to the waiting list for deceased donation and kidney allocation were split between wider societal factors and factors that influence the healthcare professional’s decision to list or accept the kidney [36, 37, 41].

Some factors influenced both the CYP’s perceived eligibility and their choice of transplant such as their knowledge around making informed choices, mental health, beliefs and coping strategies, motivation and ability to seek help, socioeconomic resources, family structure and dynamics and the community supporting them [34–45]. Racialised inequalities as a wider societal subtheme permeated across all three domains.

A narrative synthesis was performed of the remaining 40 quantitative studies, including the quantitative data embedded in the mixed method studies. Findings from these studies have been tabulated in Table 2. All studies were coded according to psychosocial factor and outcomes of transplant access.

**Table 2:**
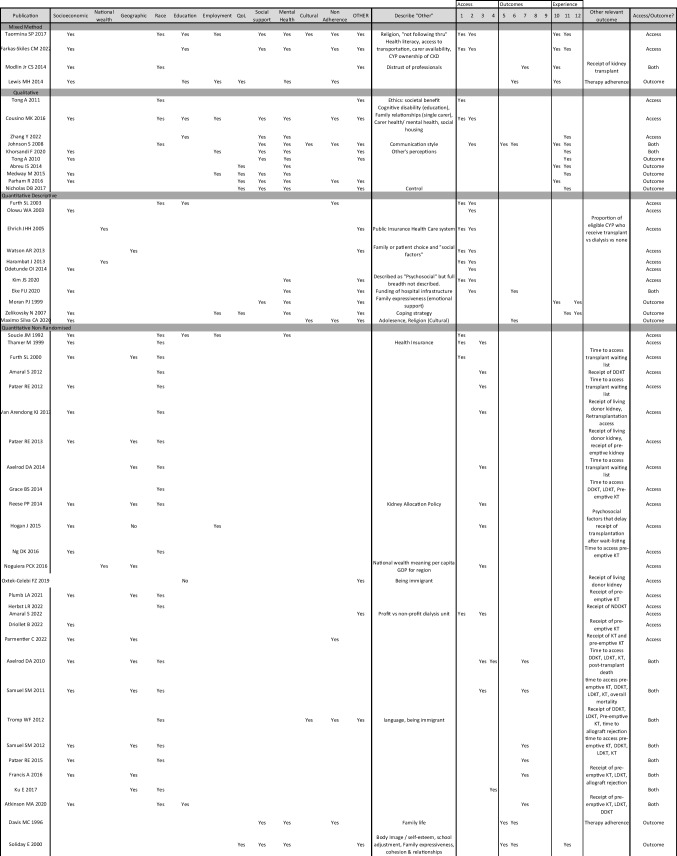

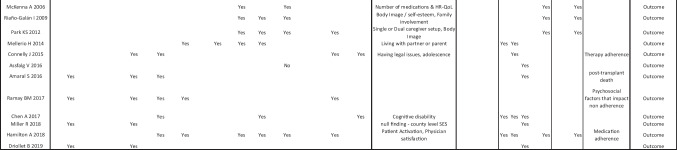
Narrative synthesis of quantitative data not included in meta-analysis

Table 3 documents whether studies in this review had concordant or different outcomes of the listed psychosocial factors having influence over transplant access, outcomes or the lived experience of CKD.

**Table 3:** Integrated analysis of psychosocial factors that are deemed to influence access to and outcomes of transplantation and lived experience of CKD

	Access			Outcome			Experience		
	Qualitative	Quantitative	Meta-analysis	Qualitative	Quantitative	Meta-analysis	Qualitative	Quantitative	Meta-analysis
Psychological/behaviour									
Mental health	Yes [36, 40, 41]				No [17]		Yes [34, 37–40, 42, 43, 45, 46]	Yes [31]	
Adjustment to CKD	Yes [36]						Yes [37–39, 42, 46]	Yes [25, 31]	
Non adherence	Yes [35, 36, 40, 41]	Yes [48]							
Beliefs	Yes [37, 40]						Yes [39, 43]		
Family choice	Yes [47]								
Motivation/follow-thru	Yes [40]								
Cognitive status	No [35]				No [15]				
Family									
Single or dual caregiver setup	Yes [35, 36, 40]							Yes [3]	
Family relationships							Yes [36, 38, 42, 43, 46]	Yes [21, 27]	
Family expressiveness (emotional support)								Yes [30]	
Family involvement							Yes [39]	Yes [29]	
Healthcare factors									
Healthcare relationships	Yes [41]						Yes [36–38, 41, 42, 46]		
Trust in healthcare	Yes [56]								
Health insurance	Yes [36, 56]	Yes and no [57, 60–64]			Yes and no [16, 20, 56, 65]		Yes [37]		
Healthcare infrastructure		Yes [19, 58]							
Social factors									
Education—available information	Yes [40]								
Education level	Yes [36, 48, 66]	Yes [67]					Yes [38]		
Health literacy		No [83]							
Education support							Yes [45]		
Education outcomes								Yes [44]	
Geography—neighbourhood wealth		Yes [7, 61, 74]			Yes and no [16, 23, 65, 75]				
Geography—distance from unit		Yes and no [47, 69–73]			Yes and no [69, 73]		Yes [34]	No [68]	
Geography—regional differences		Yes [6, 8, 9]			Yes [6]				
Geography—travel costs							Yes [38]		
Socioeconomic status	Yes [40]	Yes [7, 74, 100, 101]	Yes [60, 61, 67–71, 76]		Yes [23]	Yes [65, 69, 70, 75]	Yes [36, 38, 43]	Yes [68]	
Social support	Yes [36, 40]			Yes [35]	Yes [15]		Yes [38, 39, 42, 43, 45, 46]	Yes [22, 26]	
Housing	Yes [35, 40]								
Religion/culture	Yes [40]	Yes [54]					Yes [37]		
Societal factors									
National wealth		Yes [57, 77]							
Regional wealth		Yes [8]			No [16]				
Race	Yes [41, 56]	Yes [6, 7, 9, 48, 60, 63, 64, 66, 81]	Yes [59–61, 67–69, 72, 73, 80, 81]		Yes and no [15, 20, 56]	Yes [54, 65, 72]	Yes [37]		
Immigration status	Yes [40]	Yes and no [54, 83]			Yes and no [54]				
Kidney allocation policies	Yes [41]	Yes [9, 77, 81]							

This table summarises whether qualitative studies, quantitative studies or studies included for meta-analysis agree whether each psychosocial factor does (marked as ‘yes’) or does not (marked as ‘no’) influence access to or outcomes after transplantation or the family’s lived experience of CKD. Blank boxes indicate where no study data were found regarding the given psychosocial factor

### Psychological and behavioural factors

#### Mental health

Mental health and how well the family were adjusting to life with CKD were important aspects of living with CKD [34, 37–40, 42, 43, 45, 46]. Families reported living with stage 5 CKD as being characterised by a sense of helplessness and lack of control over their health situation [38, 39, 46]. Regaining control by developing coping strategies such as finding avenues for emotional release, becoming action-oriented and thinking positively, were reported as positive behaviours in adjusting to life with stage 5 CKD [25, 31, 37–39, 42, 45, 46].

In terms of influencing transplantation access, the above psychological and behavioural factors as well as therapy non-adherence, family decisions, motivation and ability to follow through were described [35–37, 40, 41, 47, 48]. Although poor mental health was reported to dissuade clinicians from recommending transplantation or family members viewing themselves as suitable living donors [36, 40], if the patient’s mental health was likely to deteriorate significantly due to further delays in transplantation, clinicians were inclined to expedite waitlisting [36, 41]. Cousino et al. reported one case where a CYP with intellectual disability was able to access transplantation successfully [35].

There were limited published data on the relationship between psychological and behavioural factors with transplant outcomes. One study reported that patients urgently listed due to suicidality in Europe had significantly better allograft outcomes than patients listed due to lack of dialysis access [17]. Another reported that CYP with cognitive impairment or intellectual disability had comparable transplant outcomes [15].

#### Medication non-adherence

Medication non-adherence in the post-transplant period is widely acknowledged as significantly associated with allograft rejection [49, 50] leading to further allograft loss, return to dialysis and increased mortality and morbidity [51–53]. In this review, 15 studies addressed non-adherence. Non-adherence in the pre-transplant period influenced the likelihood of clinicians recommending transplantation [35, 36, 40, 41, 48]. Given the impact non-adherence has on both transplant candidacy and outcomes, an integrated sub-analysis was undertaken of psychosocial factors associated with non-adherence (Table 4).

**Table 4:** Integrated sub-analysis of psychosocial factors that influence therapy adherence

	Qualitative		Quantitative	
	Influences therapy adherence	Does not influence therapy adherence	Influences therapy adherence	Does not influence therapy adherence
Psychological/behaviour				
Mental health	[42, 46]		[22, 44]	[18]
Adjustment to CKD			[21]	
Patient activation, CYP ownership of CKD			[22]	
Adolescence			[18, 24]	
Family				
Living with parent			[22]	
Healthcare factors				
Physician satisfaction			[22]	
Social factors				
Religion (cultural)			[24]	
Geographic			[18, 28]	[18, 28]
Education level			[22, 28]	
Having legal issues			[18]	
Social support	[37]			
Societal factors				
Immigrant status				[54]
Racism			[18, 22]	

This table summarises whether qualitative studies or quantitative studies report agreement about whether each psychosocial factor does or does not influence therapy adherence. Empty boxes indicate that this was not explored

Apart from three studies where only post-transplant adherence was examined [18, 24, 54], most studies did not differentiate between pre- or post-transplant adherence. Psychological or behavioural factors associated with barriers to adherence included poor mental health, poor adaptive functioning skills, adolescence, negative peer pressure and being areligious. Social structural factors associated with barriers to adherence included low socioeconomic status (SES), race, living a shorter distance from the transplant centre, lower maternal education level and having legal issues (i.e. involvement in court proceedings due to arrest, or incarceration) [18, 21, 22, 24, 28, 35, 37, 44, 48]. One study reported that psychosocial issues were not strongly associated with poor adherence; however, it was not clear what was categorised as a psychosocial issue in this study [18]. Conversely, greater satisfaction with their healthcare provider, living with a parent or partner (for adult-aged patients) and patient activation were significantly associated with higher medication adherence [22].

### Family dynamics

Living with CKD was perceived to stress families: caregivers spent less time with other family members, became socially restricted and financially stretched [39, 40]. In some cases, these stressors precipitated domestic upheaval and separation between partners [36, 38, 42, 46]. Family factors that ameliorated the stress of living with CKD included caregivers/parents being married, open expression of emotions and overall cohesion [3, 21, 27, 29, 30, 43, 55]. For transplant access, whether the CYP had a single or dual caregiver setup or lived in foster care influenced decisions around opting for living or deceased donation [3, 35, 36].

### Healthcare relationships and infrastructure

The interface where patients engage with healthcare can be described by health infrastructure and the relationship between clinicians and families. Families living with CKD valued feeling listened to, transparency, attentiveness and having an overall good relationship with healthcare professionals [36–38, 41, 42, 46].

Relationships between clinicians and families influenced transplant access in two ways. First, clinicians may advocate further for patients they have a good rapport with [41] and second, distrust of clinicians has been reported as a barrier for deceased organ donor registration [56].

Studies examining health infrastructure often reviewed national health insurance policies. European countries with a public health insurance system often had a higher proportion of transplanted CYP rather than those on dialysis [57]. In the context of limited national health coverage, infrastructure also influenced transplant access. In Nigeria, private funding was highly relied on for CYP to access kidney transplantation. Even with state funding, it was the private sector that performed the transplant operation [19]. Conversely in the USA, CYP receiving dialysis at a for-profit facility waited longer to be wait-listed and receive a transplant [58].

In this review, all studies examining differences between private and public health insurance, apart from one [57], were conducted in the USA. Families with private health insurance were more likely to self-report as White [56, 59, 60], had increased access to a PKT either from a deceased or living donor [61] and were more likely to be re-transplanted after first allograft failure [62]. Having any health insurance appeared to attenuate racialised health inequities faced by Hispanic CYP when accessing the waiting list. However, Hispanic CYP without any insurance still had reduced access to the waiting list overall [60]. One study complemented this finding where Hispanic families found the onerous process of applying for health insurance itself delayed their CYP’s CKD diagnosis [37]. The type of public insurance also influenced access to the waiting list. However, once on the waiting list, this had no impact on the eventual receipt of a DDKT [63]. In one study, the reliability of the family’s health insurance influenced how clinicians and families perceived a CYP’s transplant eligibility [36].

In some studies, public or private health insurance status made no difference on whether the CYP received a non-pre-emptive DDKT or LDKT, and whether they received an altruistic KT or were wait-listed for a DDKT [40, 64]. The association between allograft outcomes and health insurance status was mixed. Increased allograft failure was associated with public insurance in one study [65]. However, in other studies, public and private insurance holders had similar allograft failure rates [16, 20].

### Social factors

#### Education level and health literacy

Education featured in various ways in the lives of CYP with CKD. This included education level, the information available for families to make informed decisions, the accessibility of educational support for CYP with CKD who require it and the educational attainment of CYP as a QoL outcome measure.

An age-adjusted mixed adult and CYP study reported that having more years of formal education increased the likelihood of transplant candidacy [66]. For caregivers of CYP with CKD, higher caregiver education level improved the caregiver’s confidence in managing their CYP’s CKD [38]. The combination of higher caregiver education level and better medication adherence increased clinician recommendation of transplant candidacy in CYP [36, 48]. One study reported that CYP whose mothers had tertiary education were more likely to receive pre-emptive transplantation [67].

Reduced health literacy or access to health information has been cited as a barrier to living donation [40]. Educational attainment has been used as a transplant outcome measure. Paediatric transplant recipients in adulthood were reported to have lower educational attainment than the general population [26] and adult transplant recipients [44]. One study found that CYP with CKD faced challenges in accessing educational support [45].

#### Geographic factors

Where the CYP lived in terms of location within their country, distance from their kidney unit and neighbourhood wealth could influence their experience of living with CKD. However, direct comparisons of studies were not possible due to the heterogeneity of financial resources and transportation infrastructure between regions.

Geographic remoteness of the CYP’s home from their nearest kidney unit was not associated with late presentation of stage 5 CKD in the UK [68]. However, from a day-to-day perspective of living with CKD, having to budget for travel costs influenced the family’s choice of dialysis modality [38] and even convinced them to move closer to the kidney unit [34].

When it comes to transplantation access, regional variation has been observed within countries [6–9]. Most studies agreed that geographic remoteness influenced access to transplantation [47, 69–73]. European studies have shown that CYP living in non-urban or remote areas were more likely to opt for PKT [47, 71]. This association was reversed in Australia [70]. However, there was no significant association between the CYP’s geographic remoteness in Canada and choosing PKT [73]. CYP living remotely were more likely to opt for LDKT and less likely to access DDKT even after waitlisting [69, 73]. Neighbourhood affluence was reported to increase access to PKT in the USA [61, 65] and overall transplantation access in France [74].

The association between geographic variables and transplant outcomes was mixed and dependent on how the variable was defined. For example, one USA study found no association between county-defined SES characteristics and paediatric KRT outcomes [16] whereas transplant regions with the highest transplantation listings and rates had significantly reduced mortality rates in the USA [6]. Another study reported that, compared with White and Hispanic CYP, there was a higher risk of death for Black CYP on KRT living in the West, Midwest and Northeast of the USA, but not in the South [23]. Patients living remotely in the USA had an increased risk of post-transplant death and a borderline lower risk of DDKT allograft failure [69]. However in Canada, geographic remoteness had no significant relationship with allograft failure [73].

Living in a deprived neighbourhood in the USA made no difference to allograft failure among pre-emptive transplant recipients, but increased allograft failure risk for non-PKT recipients [65]. Similar findings have been reported in France, where allograft failure risk increased for those living in deprived neighbourhoods [75].

#### Socioeconomic status

**Fig. 3:**
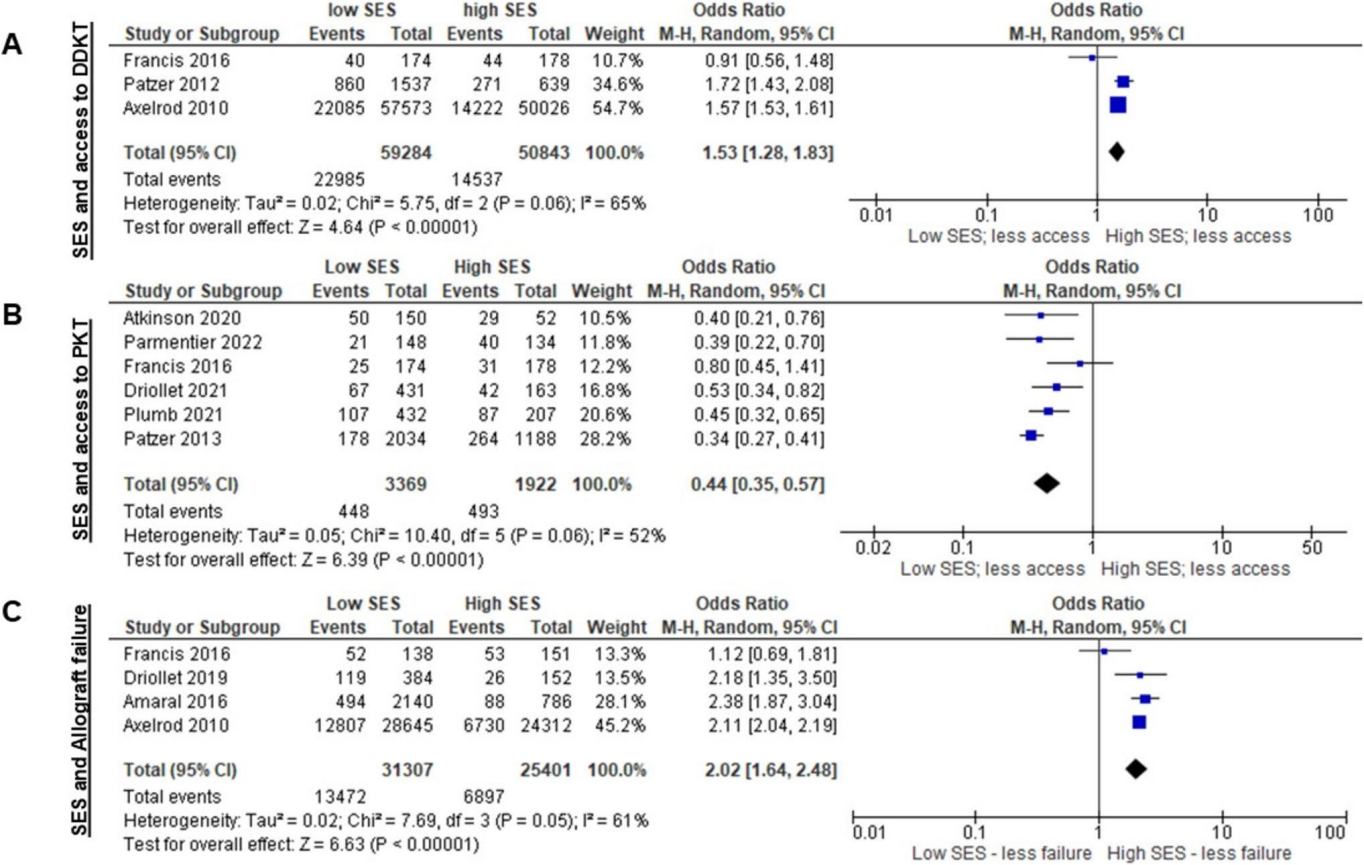
Forest plots of meta-analyses for socioeconomic status and transplant access and outcomes

Socioeconomic deprivation can influence how available different transplant modalities are to families. Meta-analysis showed that families experiencing lower SES as a potential stressor were more likely to access a DDKT (Fig. 3A) [60, 69, 70] and less likely to access PKT (Fig. 3B) [61, 67, 68, 70, 71, 76]. A meta-analysis for accessing an LDKT could not be performed due to a lack of eligible studies. When reviewing the influence SES had on allograft outcomes in the meta-analysis in Fig. 3C, those from a lower SES background experienced greater allograft failure risk [65, 69, 70, 75].

Families reported that managing their CYP’s CKD was time-consuming and reduced the caregiver’s ability to work. Given the added costs of living with CKD such as travel, there was an advantage to being more financially secure or having a flexible employer [36, 38, 43]. Barriers to LDKT reported by families included financial and employment-related issues such as medical leave being unpaid or job insecurity after kidney donation [40], which supports the association between lower SES and increased DDKT access in the meta-analysis.

#### Social support

There is no defined term for ‘social support’ as a quantifiable variable. Therefore, we have included support from friends, extended family, school, employers, work colleagues, hospital personnel and government-issued benefits.

From a day-to-day perspective, living with CKD was seen as isolating. Families felt misunderstood or discriminated against by others in their social network [26, 39, 42, 45, 46]. Caregivers described a ‘burden of sole responsibility’ and were anxious about relinquishing their CYP’s care even when support was available [38]. Fathers of CYP with CKD were described as particularly reluctant to seek support [39]. Some viewed government support as a last resort and difficult to obtain [38]. Families who wanted more psychological support reported challenges in accessing it [45]. Caregivers reported that mental stresses imposed by their social network made adapting to life with CKD difficult [43]. It was generally agreed that having a supportive network through friends, schools, hospital personnel, extended families, work colleagues and other families living with CKD positively impacted the experience of living with CKD [22, 42, 43, 45]. Moreover, the availability of ‘back up carers’ [36] and general social support influenced candidate eligibility and LDKT availability [40].

#### Other social factors

Other social factors included housing and cultural or religious beliefs. Housing status, eviction and homelessness influenced both LDKT availability [40] and candidate eligibility [35]. Cultural beliefs that differed from biomedical practice and Western values sometimes delayed families from seeking formal healthcare [37].

### Wider societal factors

#### National and regional wealth

There were no data on how national and regional wealth influenced a family’s experience of living with CKD. However, Europe-wide studies investigating the variation in the delivery of paediatric nephrology care demonstrated a persistent association between kidney transplant rates and national wealth—measured either as gross national product (GNP) or gross domestic product (GDP) [57, 77]. This finding appeared consistent on a regional level within countries. A Brazilian study reported that regional economic wealth was associated with better access to kidney transplantation [8]. Conversely, a USA study found no correlation between county-level SES rankings with county-level transplant outcomes [16].

#### Racialised inequities

Race is now widely acknowledged to be a social construct, derived from sociopolitical arrangements that advantage or disadvantage groups based on their perceived racial identity, ethnicity or cultural heritage. Where studies reported outcomes by race or ethnicity, we present these findings as reported by the authors. Consistent with contemporary health equity frameworks, we interpret race not as a biological variable but as a proxy for exposure to racism and structural inequities that shape health outcomes [78, 79]. Studies included in this review reported differences between racially minoritised (Black, Hispanic, Indigenous) and racially centred (White) groups when accessing healthcare and transplantation. Although none of the included studies specifically named ‘racism’ in their reports, some studies alluded to racially driven bias or barriers and structurally based inequities to explain the observed differences between racially minoritised and centred groups [20, 48, 56, 60, 61, 68, 72, 80]. Hispanic CYP experienced a more circuitous route to CKD care than White CYP [37]. Black CYP were less likely to be recommended as a transplant candidate than White CYP [66]. Parental education partially mediated these outcomes: Black CYP whose parents had higher educational attainment were more likely to be recommended for transplantation than Black CYP whose parents had lower educational attainment [48].

**Fig. 4:**
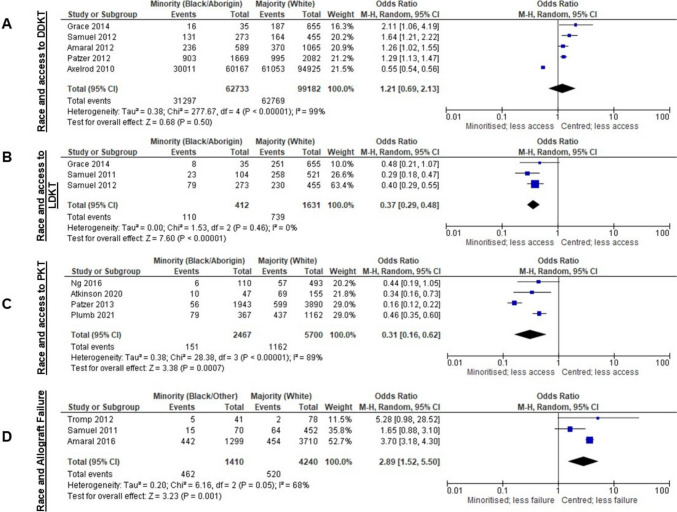
Forest plots of meta-analyses for race and transplant access and outcomes

In meta-analysis, being racially minoritised did not significantly affect deceased donor kidney transplantation (DDKT) access [60, 69, 73, 80, 81] (Fig. 4A). However, studies not included for meta-analysis reported that CYP from racially minoritised groups were less likely to be placed on the waiting list [6, 7, 63] and less likely to receive a DDKT after waitlisting [6, 9]. Qualitative findings reinforced these inequities: clinicians voiced concern that allocation policies heavily reliant on tissue typing disadvantage racially minoritised groups [41], while members of the Black community in the USA reported mistrust in clinicians as a barrier to registering as a DDKT organ donor [56].

Access to LDKT (Fig. 4B) [72, 73, 80] and PKT (Fig. 4C) [59, 61, 67, 82] was lower for racially minoritised CYP. Results of studies not included in meta-analyses agreed with these findings [6, 20, 40, 62]. Furthermore, White CYP were more likely to receive altruistic non-related LDKT [64].

By 5 years, racially minoritised groups had higher allograft failure risk (Fig. 4D) [54, 65, 72]. The breakdown of this was more nuanced in the studies not included for meta-analysis. Black CYP who were DDKT recipients had more HLA mismatches, had longer cold ischaemia time and were more likely to experience delayed graft function and allograft rejection than White DDKT recipients [15, 20, 56]. However, for LDKT, HLA mismatching and allograft rejection were comparable across different racial groups [56].

Studies that included both SES and racial data found the effects overlapped. In one study, once the data had been adjusted for SES and other confounders, the association between reported race and access to the waiting list was attenuated for Black CYP and eliminated for Hispanic CYP. However, racialised health inequities persisted in the young adult cohort (18–20 years), suggesting that other structural factors were involved [60]. These studies underscore the intersectional nature of inequities, where racism, SES, education and healthcare access interact rather than operate independently.

#### Immigrant status

Studies included in this review described immigration status as a social factor, but none clarified whether all migrants were included such as refugees or asylum seekers. Immigration status was listed by families as a barrier to LDKT donation in the USA [40]. This picture was mixed in Europe: a study in Austria reported no difference in LDKT or PKT donation rates between immigrant and non-immigrant families [83], whereas a study in the Netherlands and Belgium reported that immigrant families received fewer LDKT than non-immigrant families [54]. Non-immigrant CYP had a longer rejection-free period post-transplantation in the Netherlands and Belgium compared with CYP from immigrant families. However, there was no difference between non-immigrant and immigrant CYP in suspected medication non-adherence and allograft function deterioration based on eGFR during the study period [54].

#### Kidney allocation policies

Kidney allocation policies are typically designed to ethically maximise societal benefit and equity. Therefore, issues around kidney allocation policies such as unit behaviours in accepting or rejecting a kidney as well as the policies themselves have an impact on DDKT access. Regarding decision-making around waitlisting and accepting deceased donor organs, paediatric nephrologists reported a desire to advocate for their patient, maintain their professional integrity and protect their kidney unit’s reputation [41].

In Europe, regions with kidney allocation policies that included paediatric priority had a significantly decreased waiting time for DDKT and an increased transplantation rate among CYP. Regions with paediatric priority had a lower proportion of LDKT compared with regions without [77]. However, not all kidney allocation policies with paediatric priority resulted in increased equity for everyone. In the USA, the SHARE-35 policy was implemented in 2005. This policy had paediatric priority, but in exempting circumstances, kidneys may be diverted to adult recipients. Some regions with longer DDKT waiting times had a lower ratio of kidneys to paediatric candidates and more diversions of kidneys to adult recipients [9]. Furthermore, there was a shift from LDKT to DDKT, and racialised health inequities persisted since rolling out the SHARE-35 policy [81].

## Discussion

Existing literature reviews examine psychosocial factors relating to kidney transplantation, living donor experiences and CKD without KRT [84–89]. None of these reviews include paediatric cohorts, nor do they examine psychosocial factors that influence access to and outcomes after kidney transplantation. Our review analysed 66 studies that examined these psychosocial factors including the lived experience of families living with CKD. All factors were reviewed through thematic synthesis or narrative synthesis depending on whether the data were qualitative or quantitative, and 13 eligible studies were examined through meta-analysis. Through its broad inclusion of quantitative, qualitative and mixed-method study data, our unique review describes not only the wide breadth of psychosocial factors that impact a CYP’s lived experience of CKD, their access to and outcomes after transplantation, but also integrates these findings and provides a more nuanced understanding of this field.

To broadly summarise our findings: living with CKD has a multifaced impact on families, and their psychosocial contexts influenced their transplant access and outcomes. Better transplant access and outcomes were associated with better mental health support from family, social network and health professionals, higher SES, higher education, greater regional or national wealth, sufficient health insurance coverage and belonging to a racially centred group (e.g. self-identifying as White in the USA). However, the reality of our findings was more layered and did not necessarily follow linear associations. Poor mental health could hinder both transplant recipient and donor candidacy, but conversely, if further transplant delays were thought likely to trigger a substantial deterioration in mental health, waitlisting could be expedited [17, 41]. Most studies agreed that geographical remoteness influenced transplant access, but the direction of its influence differed between regions [47, 70, 71]. Racialised health inequities observed in this review did not uniformly manifest across all minoritised groups, and the health inequities interacted with other factors such as SES and education [48, 60].

How can these psychosocial factors be addressed? The psychosocial experience of living with CKD is complex. Families face stressors from the added costs of living with CKD, difficulties with financial and employment security, social isolation and poorer mental health [34, 35, 38–40, 42, 43, 45, 46]. In some cases, these stressors can lead to complete upheaval within the family, for example the separation of parents or the need to relocate closer to the treating centre [34, 38, 42]. However, the challenges of living with CKD could be mitigated by strategies to enhance family cohesion, better education, a supportive social network and the fostering of more positive or action-oriented coping strategies [30, 48, 55, 66, 67]. These could be the focus of targeted interventions by health and social care professionals to improve family experience. Family therapists and clinical psychologists can help address issues around family cohesion, mental health and coping strategies [90]. Social workers can direct families to financial support they may be eligible for. Improved information through written communication, other multimedia sources and play preparation can improve education [91]. The wider paediatric kidney multi-disciplinary team (doctors, nurses, dieticians, pharmacists, youth workers and others) can offer further practical and emotional support through their professional roles and relationships with families [91]. Finally, families can be signposted to peer support groups, which can attenuate social isolation and provide further emotional support [91].

The psychosocial factors that define the lived experience of CKD are interwoven with factors that influence access to transplantation and its outcomes. The deemed eligibility of the CYP for transplantation is often based on whether the family’s current adjustment to living with CKD can predict how well they will cope and thrive post-transplant [36, 41]. Therapy adherence to transplantation is critical in preventing allograft failure. Unsurprisingly, the psychosocial factors that influenced adherence, transplant outcomes and the lived experience of CKD were also described among factors that influenced access [21, 22, 24, 28, 35, 37, 44, 48]. These factors included mental health, coping strategies, socioeconomic resources, social network, education and family structure—some of which can be targeted by health and social care professionals. Although the need for robust psychosocial support for CYP with stage 5 CKD is widely acknowledged, there is great variability in provision of these services [92, 93].

However, there are wider factors at play that cannot be pinned to the individual CYP and their family. These include the relationship with their clinicians and other structural determinants of health that drive social inequalities—such as access to advantageous health insurance, national health coverage policies, geographic location, organ allocation policies, immigration status, structural racism and national wealth—demonstrating health inequities around paediatric kidney transplant are not just at an individual level but also at a national and international levels.

Moreover, these societal factors demonstrate a complex interplay between themselves rather than behaving as additive single factors, revealing a more nuanced picture regarding transplant access inequities. This was especially apparent in studies reporting racialised health inequities. Although ‘racism’ as a term was not explicitly used by study authors, they alluded to this more nuanced picture by referring to structural drivers of these inequities rather than inherent biological differences. Racism operates at multiple levels—structural (policies and allocation criteria), institutional (healthcare practices) and interpersonal (bias and discrimination)—to disadvantage racially minoritised CYP. For example, allocation systems reliant on tissue typing disadvantage certain groups due to population-level genetic variation, a structural inequity embedded in policy. Intersectionality, first articulated by Crenshaw, highlights how systems of oppression overlap in ways that are not simply additive [94, 95]. For example, Black CYP with lower parental education were less likely to be recommended for transplantation than those with higher parental education, reflecting the compounded effects of racism, education and SES [7, 60].

An individual’s subjective perception of stress or their place in society, in lieu of their social background (i.e. race or socioeconomic status), can influence health outcomes [96, 97]. Therefore, strategies tackling these societal factors require changes at all tiers—from the individual to the wider structures that shape society. For example, racialised health inequities can be tackled by educating professionals on implicit or explicit racial bias, encouraging diversity at a recruitment level and reviewing wider policies and practices that disadvantage minoritised groups [98]. The way racialised health inequities are reported can also play a role in addressing racial bias. Several included studies framed racially minoritised patients as less adherent or less motivated. This ‘deficit framing’ risks obscuring the role of systemic barriers, such as limited access to healthcare infrastructure, clinician mistrust or bias. Reframing inequities in terms of barriers rather than deficits is critical to avoiding stigma and promoting structural solutions. However, this is easier said than done. If change does not align across all social structures, the health inequities gap can widen even further [99]. Further studies are needed to understand these wider societal-level factors to better address the health inequities driven by them.

### Strengths and limitations

One strength in this review was the rigor in the study selection and study quality assessment processes by ensuring two researchers were involved in the initial selection or assessment, and consensus was achieved through a third researcher. Another strength was its inclusivity with respect to study design and therefore the comprehensive breadth of psychosocial factors that could be captured. However, the heterogeneity of study design, independent variables and outcome measures limited their comparability. As demonstrated in Table 2, none of the studies utilised the same validated psychosocial measure, and only two psychosocial factors (racialised and SES-related health inequities) were suitable for meta-analysis. Therefore, the strength of this review’s findings, where meta-analysis was not possible, is limited.

At least 12 (18%) of the included studies were dated, with published findings between 1992 and 2010 [7, 21, 25, 27, 29, 31, 37, 48, 57, 63, 66, 100]. Given the medical progress in CKD management since the 1990 s and the weight social context has on psychosocial studies, the applicability of some of these study findings to the present day may be limited. Despite this, findings from all 12 studies were congruent with the more recent studies in this review. Although this confirms the continued relevance of their findings, it is also a sobering reflection of the limited progress made in addressing health inequities driven by psychosocial factors.

Interpretation of some findings was bounded by data from adult–paediatric studies. For example, the meta-analysis on SES included a study with a sizeable mixed adult–paediatric cohort, where a more granular breakdown of its paediatric data was not possible [69].

Another limitation of the meta-analyses was the inclusion of studies from different nations and therefore with differing societal structures (e.g. health policy, law, culture) that would drive structural racism or SES-related health inequalities. Caution in interpreting the findings is therefore necessary as this analysis may inadvertently, for example, compare transplant access between differing national healthcare policies rather than the influence SES has on an individual.

Furthermore, the meta-analysis on racialised health inequities was limited to comparing one racially centred group (White) against all racially minoritised groups (Black, Hispanic, Indigenous). Although this meta-analysis identifies racialised inequities around transplant access and outcomes overall, it cannot represent the nuanced experience of each group and every region. Depending on the societal context, different advantages and disadvantages may be experienced by different racially minoritised groups—such as the degree to which accounting for SES attenuates the effect race has for Black and Hispanic CYP [60]. Unfortunately, there were insufficient studies to examine the experience of each group in a meta-analysis.

Interpretation of results regarding immigration status was also restricted by the heterogeneous definition of immigrant between the included studies. One study defined natives as CYP of Western European origin and immigrants as CYP one or both of whose parents were born in non-Western European countries [54]. Another study compared Austrian CYP with CYP whose families originated from outside Austria [83]. None of the included studies indicated whether immigrant CYP included refugees or asylum-seekers, who may have experiences unique from other migrant families.

Compared with studies examining access and the lived experience of CKD, there was a paucity of study data on transplant outcomes. For instance, there were no studies that directly examined the association between transplant outcomes with family factors, relationship with healthcare workers, education or religious and cultural beliefs.

### Conclusions and implications of key findings

This systematic review has highlighted the wide breadth of psychosocial factors that influence the lived experience of CKD and access to and outcomes after transplantation. There is an unsurprising overlap between these factors and psychosocial factors influencing therapy adherence. Some psychosocial factors identified by this review can be targeted and managed by health and social care professionals to improve transplant access and outcomes. However, there are wider societal-level factors that are outside the remit of the individual health or social care professional that need to be addressed through changes in national infrastructures, policies and wider cultural perspectives. Further studies are needed to better understand the complex relationship between these psychosocial factors and kidney transplantation outcomes.

## Supplementary information

Below is the link to the electronic supplementary material.

Graphical abstract (PPTX 77.7 KB)

ESM 2 (PDF 163 KB)

ESM 3 (PDF 775 KB)

ESM 4 (PDF 100 KB)

## Data Availability

The datasets generated and analysed during the current study are available from the corresponding author on reasonable request.

## Declarations

### Disclaimer

The views expressed are those of the author(s) and not necessarily those of the NHS, the NIHR or the Department of Health and Social Care.
