# Supplementary material for: The psychosocial factors influencing paediatric kidney transplantation access, their outcomes and the patient and family’s perceived quality of life: a systematic review and meta-analysis
Source: Pediatr Nephrol. 2025 Dec 10;41(7):2001–20. doi: 10.1007/s00467-025-07058-9 (PMC13197345; doi:10.1007/s00467-025-07058-9)
Supplement: Supplementary file 1 — Graphical abstract (PPTX 77.7 KB) [file 467_2025_7058_MOESM1_ESM.pptx]

## Slide 1
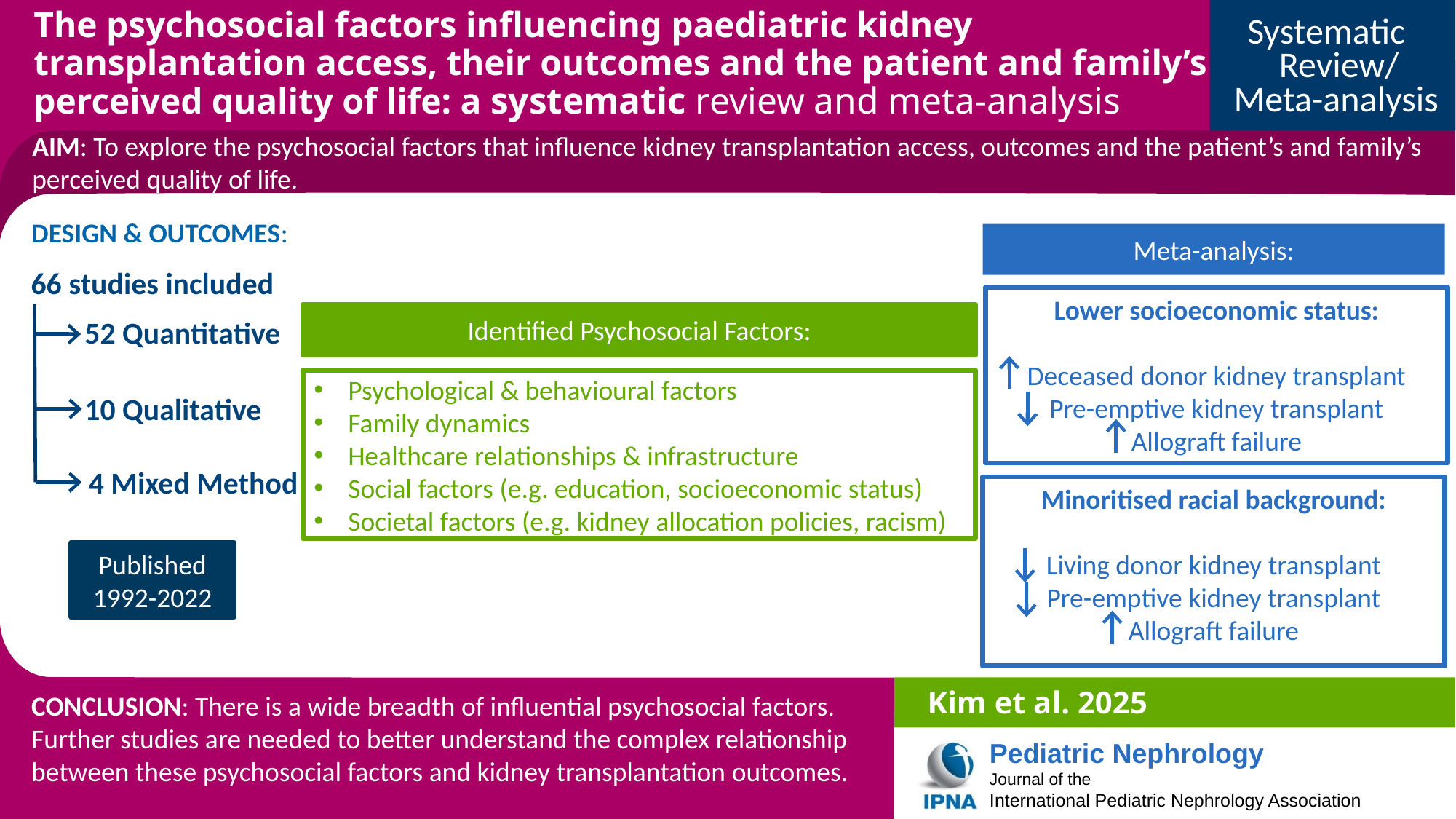

The psychosocial factors influencing paediatric kidney transplantation access, their outcomes and the patient and family’s perceived quality of life: a systematic review and meta-analysis
AIM: To explore the psychosocial factors that influence kidney transplantation access, outcomes and the patient’s and family’s perceived quality of life.
DESIGN & OUTCOMES:
Meta-analysis:
66 studies included
Lower socioeconomic status:
Deceased donor kidney transplant
Pre-emptive kidney transplant
Allograft failure
Identified Psychosocial Factors:
52 Quantitative
Psychological & behavioural factors
Family dynamics
Healthcare relationships & infrastructure
Social factors (e.g. education, socioeconomic status)
Societal factors (e.g. kidney allocation policies, racism)
10 Qualitative
4 Mixed Method
Minoritised racial background:
Living donor kidney transplant
Pre-emptive kidney transplant
Allograft failure
Published
1992-2022
Kim et al. 2025
CONCLUSION: There is a wide breadth of influential psychosocial factors. Further studies are needed to better understand the complex relationship between these psychosocial factors and kidney transplantation outcomes.
