# Supplementary material for: The psychosocial factors influencing paediatric kidney transplantation access, their outcomes and the patient and family’s perceived quality of life: a systematic review and meta-analysis
Source: Pediatr Nephrol. 2025 Dec 10;41(7):2001–20. doi: 10.1007/s00467-025-07058-9 (PMC13197345; doi:10.1007/s00467-025-07058-9)
Supplement: Supplementary file 2 — (PDF 163 KB) [file 467_2025_7058_MOESM2_ESM.pdf]

What are the psychosocial factors that influence access to kidney transplantation (and their outcomes) for children and young people?

Search Strategy

Search Strategy

1. Years considered: 1st January 1964 till 30th November 2022
2. Language: English Only

Key Principles behind Search Terms:

1. **Include** (kidney transplant* OR renal transplant* OR renal allograft OR kidney allograft OR renal replacement therapy OR dialysis)
2. **AND** (child* or paediatr* or pediatr* or young pe* or adoles* or teen* or infan* or juveni* or preschool* or pre-school* or youth* or schoolchild* or neonat* or newborn* or baby or babies)
3. **AND** (psych* OR anxiety OR depression OR adheren* OR complian* OR concord* OR social OR emotional OR experience OR beliefs OR perception* OR quality of life OR family function* OR coping OR body-image OR body image OR self-esteem OR adjustment OR socioecon* OR socio-econ* OR attitude OR interpersonal relat* OR inter-personal relat*)
4. **AND** (access OR list* OR waitlist* OR wait-list)

Search Strategy for MEDLINE

1. Exp renal replacement therapy/ or exp renal dialysis/ or kidney transplantation/
2. (dialysis OR renal replacement therapy).mp. [mp=title, abstract, original title, name of substance word, subject heading word, floating sub-heading word, keyword heading word, organism supplementary concept word, protocol supplementary concept word, rare disease supplementary concept word, unique identifier, synonyms]
3. ((kidney or renal) adj2 (disease or failure or insufficiency or transplant* or allograft*)) adj2 (chronic or established or terminal or end-stage) .mp. [mp=title, abstract, original title, name of substance word, subject heading word, floating sub-heading word, keyword heading word, organism supplementary concept word, protocol supplementary concept word, rare disease supplementary concept word, unique identifier, synonyms]
4. adolescent medicine/ or exp pediatrics/
5. adolescent/ or exp child/ or exp infant/
6. (child* or paediatr* or pediatr* or young pe* or adoles* or teen* or infan* or juveni* or preschool* or pre-school* or youth* or schoolchild* or neonat* or newborn* or baby or babies).mp. [mp=title, abstract, original title, name of substance word, subject heading word, floating sub-heading word, keyword heading word, organism supplementary concept word, protocol supplementary concept word, rare disease supplementary concept word, unique identifier, synonyms]
7. "Quality of Life"/
8. exp "Treatment Adherence and Compliance"/
9. exp Emotions/
10. depression/ or exp stress, psychological/ or exp child behavior/

11. exp Patient-Centered Care/
12. exp Interpersonal Relations/
13. exp Socioeconomic Factors/
14. exp Psychology/
15. exp sociology/ or exp mental health services/
16. (psych* OR anxiety OR depression OR adheren* OR complian* OR concord* OR social OR emotional OR experience OR beliefs OR perception* OR quality of life OR family function* OR coping OR body-image OR self-esteem OR adjustment OR socioecon* OR socio-econ* OR attitude OR interpersonal relat* OR inter-personal relat*).mp. [mp=title, abstract, original title, name of substance word, subject heading word, floating sub-heading word, keyword heading word, organism supplementary concept word, protocol supplementary concept word, rare disease supplementary concept word, unique identifier, synonyms]
17. 1 or 2 or 3
18. 4 or 5 or 6
19. 7 or 8 or 9 or 10 or 11 or 12 or 13 or 14 or 15 or 16
20. (Access or list* or waitlist*) .mp. [mp=title, abstract, original title, name of substance word, subject heading word, floating sub-heading word, keyword heading word, organism supplementary concept word, protocol supplementary concept word, rare disease supplementary concept word, unique identifier, synonyms]
21. 17 AND 18 AND 19 AND 20
22. Limit 20 to English language
23. Limit 22 to yr=”1964 – 2021”

Search Strategy for PsychInfo

1. Adolescent medicine/ or exp pediatrics/
2. (child* or paediatr* or pediatr* or young pe* or adoles* or teen* or infan* or juveni* or preschool* or pre-school* or youth* or schoolchild* or neonat* or newborn* or baby or babies).mp. [mp=title, abstract, heading word, table of contents, key concepts, original title, tests & measures, mesh]
3. “Quality of Life”/
4. Exp Emotions/
5. Depression/ or exp stress, psychological/ or exp child behavior/
6. Exp Psychology/
7. Exp sociology/ or exp mental health services/
8. Exp parent child relations/
9. exp adolescent attitudes/ or exp adolescent behavior/ or exp adolescent health/ or exp adolescent psychology/
10. exp Child Attitudes/ or exp Anxiety/ or exp Academic Achievement/
11. exp sociocultural factors/ or exp socioeconomic status/
12. (psych* OR anxiety OR depression OR adheren* OR complian* OR concord* OR social OR emotional OR experience OR beliefs OR perception* OR quality of life OR family function* OR coping OR body-image OR self-esteem OR adjustment OR socioecon* OR socio-econ* OR attitude OR interpersonal relat* OR inter-personal relat*).mp. [mp=title, abstract, original title, name of substance word, subject heading word, floating sub-heading word, keyword heading word, organism supplementary concept word, protocol supplementary concept word, rare disease supplementary concept word, unique identifier, synonyms]

13. (dialysis OR renal replacement therapy).mp. [mp=title, abstract, original title, name of substance word, subject heading word, floating sub-heading word, keyword heading word, organism supplementary concept word, protocol supplementary concept word, rare disease supplementary concept word, unique identifier, synonyms]
14. ((kidney or renal) adj2 (disease or failure or insufficiency or transplant* or allograft*)) adj2 (chronic or established or terminal or end-stage) .mp. [mp=title, abstract, original title, name of substance word, subject heading word, floating sub-heading word, keyword heading word, organism supplementary concept word, protocol supplementary concept word, rare disease supplementary concept word, unique identifier, synonyms]
15. Organ transplantation/
16. Exp dialysis/
17. 1 OR 2
18. 3 OR 4 OR 5 OR 6 OR 7 OR 8 OR 9 OR 10 OR 11 OR 12
19. 13 OR 14 OR 15 OR 16
20. (Access or list* or waitlist*) .mp. [mp=title, abstract, original title, name of substance word, subject heading word, floating sub-heading word, keyword heading word, organism supplementary concept word, protocol supplementary concept word, rare disease supplementary concept word, unique identifier, synonyms]
21. 17 AND 18 AND 19 AND 20
22. Limit 21 to English language
23. Limit 22 to yr="1964 – 2021"

Search Strategy for CINAHL

Expanders - Apply equivalent subjects

Search modes - Boolean/Phrase

Limiters - Published Date: 19640101-20201231; English Language

1. dialysis OR renal replacement therapy
2. ((kidney or renal) N1 (disease or failure or insufficiency or transplant* or allograft*)) N1 (chronic or established or terminal or end-stage)
3. (MH "Kidney Transplantation+") OR (MH "Dialysis+") or (MH "Renal Replacement Therapy+") OR (MH "Kidney Failure, Chronic+") OR (MH "Renal Insufficiency, Chronic+")
4. S1 OR S2 OR S3
5. child* or paediatr* or pediatr* or "young people" or "young person" or adoles* or teen* or infan* or juveni* or preschool* or "pre-school*" or youth* or schoolchild* or neonat* or newborn* or baby or babies
6. (MH "Child+") OR (MH "Infant+") OR (MH "Pediatric Care+") OR (MH "Adolescent Psychology") OR (MH "Adolescent Psychiatry") OR (MH "Adolescent Medicine") OR (MH "Adolescent Health") OR (MH "Adolescent Coping Orientation for Problem Experiences") OR (MH "Adolescence+") OR (MH "Child, Preschool") OR (MH "Infant, Newborn+") OR (MH "Infant Behavior")
7. 5 OR 6
8. psych* OR anxiety OR depression OR adheren* OR complian* OR concord* OR social OR emotional OR experience OR beliefs OR perception* OR "quality of life" OR "family function*" OR coping OR "body-image" OR "self-esteem" OR adjustment OR socioecon* OR "socio-econ*" OR attitude OR "interpersonal relat*" OR "inter-personal relat*"

9. (MH "Psychology+") OR (MH "Child Psychology") OR (MH "Adaptation, Psychological+") OR (MH "Psychology, Social+") OR (MH "Psychology, Educational+") OR (MH "Psychology, Developmental") OR (MH "Psychology, Clinical+") OR (MH "Psychology, Applied+") OR (MH "Health Psychology") OR (MH "Conflict (Psychology)+") OR (MH "Emotional Maturity") OR (MH "Social Isolation+") OR (MH "Medication Compliance") OR (MH "Depression+") OR (MH "Life Experiences+") OR (MH "Social Adjustment") OR (MH "Anxiety+") OR (MH "Quality of Life+") OR (MH "Body Image+") OR (MH "Family Functioning+")
10. 8 OR 9
11. Access OR list* OR waitlist*
12. 4 AND 7 AND 10 AND 11

Search Strategy for Web of Science

- AND LANGUAGE: (English)
- Indexes=SCI-EXPANDED, SSCI, A&HCI, CPCI-S, CPCI-SSH, BKCI-S, BKCI-SSH, ESCI, CCR-EXPANDED,
- IC Timespan=1964-2020

1. TS=(dialysis OR “renal replacement therapy”)
2. TS=(kidney or renal) NEAR1 (disease or failure or insufficiency or transplant* or allograft*) NEAR1 (chronic or established or terminal or “end-stage”)
3. KP=(dialysis OR “renal replacement therapy”)
4. KP=(kidney or renal) NEAR1 (disease or failure or insufficiency or transplant* or allograft*) NEAR1 (chronic or established or terminal or “end-stage”)
5. #1 OR #2 OR #3 OR #4
6. TS=(psych* OR anxiety OR depression OR adheren* OR complian* OR concord* OR social OR emotional OR experience OR beliefs OR perception* OR “quality of life” OR “family function*” OR coping OR “body-image” OR “body image” OR “self-esteem” OR adjustment OR socioecon* OR “socio-econ*” OR attitude OR “interpersonal relat*” OR “inter-personal relat*”)
7. KP=(psych* OR anxiety OR depression OR adheren* OR complian* OR concord* OR social OR emotional OR experience OR beliefs OR perception* OR “quality of life” OR “family function*” OR coping OR “body-image” OR “body image” OR “self-esteem” OR adjustment OR socioecon* OR “socio-econ*” OR attitude OR “interpersonal relat*” OR “inter-personal relat*”)
8. #6 OR #7
9. TS=(child* or paediatr* or pediatr* or "young person" or "young people" or adoles* or teen* or infan* or juveni* or preschool* or "pre-school*" or youth* or schoolchild* or neonat* or newborn* or baby or babies)
10. KP=(child* or paediatr* or pediatr* or "young person" or "young people" or adoles* or teen* or infan* or juveni* or preschool* or "pre-school*" or youth* or schoolchild* or neonat* or newborn* or baby or babies)
11. #10 OR #11
12. TS=(access or list* or “waitlist*”)
13. #5 AND #8 AND #11 AND #12
