# Supplementary material for: The psychosocial factors influencing paediatric kidney transplantation access, their outcomes and the patient and family’s perceived quality of life: a systematic review and meta-analysis
Source: Pediatr Nephrol. 2025 Dec 10;41(7):2001–20. doi: 10.1007/s00467-025-07058-9 (PMC13197345; doi:10.1007/s00467-025-07058-9)
Supplement: Supplementary file 4 — (PDF 100 KB) [file 467_2025_7058_MOESM4_ESM.pdf]

Validated psychosocial outcome measures utilised by included studies

Author / Year	Setting	Population	Measure
Davis 1996	USA	5-18 years	• Vineland Adaptive Behaviour Scales<br>• Family Relations Index<br>• Primary Communication Inventor<br>• Patient Adherence Evaluation Form (Physician)<br>• PAEF (Dietician)<br>• PAEF (Appointment Clerk)
Soliday 2000	USA	2-18 years	• Family Environment Scale<br>• Child Behaviour check-list<br>• Parenting Stress Index Form Short Form
McKenna 2006	Canada	2-18 years	• PedSQL 4.0 Core
Riaño-Galán I 2009	Spain	10-21 years	• Child Health and Illness Profile (Adolescent)
Park 2012	S Korea	2-18 years	• PedSQL 3.0 ESRD
Hamilton 2018	UK	16-30 years	• Warwick-Edinburgh Mental Wellbeing Scale<br>• Morisky Medication Adherence Scale
Maximo Silva CA 2020	Brazil	3-17 years	• Basel Assessment of Adherence to Immunosuppressive Medication Scale (BAASIS)
